# Propagating semantic information in biochemical network models

**DOI:** 10.1186/1471-2105-13-18

**Published:** 2012-01-30

**Authors:** Marvin Schulz, Edda Klipp, Wolfram Liebermeister

**Affiliations:** 1 Institut für Biologie, Theoretische Biophysik, Humboldt-Universität zu Berlin, Invalidenstr. 42, 10115 Berlin, Germany; 2 Institut für Biochemie, Charité-Universitätsmedizin Berlin, Seestr. 73, 13347 Berlin, Germany

## Abstract

**Background:**

To enable automatic searches, alignments, and model combination, the elements of systems biology models need to be compared and matched across models. Elements can be identified by machine-readable biological annotations, but assigning such annotations and matching non-annotated elements is tedious work and calls for automation.

**Results:**

A new method called "semantic propagation" allows the comparison of model elements based not only on their own annotations, but also on annotations of surrounding elements in the network. One may either propagate feature vectors, describing the annotations of individual elements, or quantitative similarities between elements from different models. Based on semantic propagation, we align partially annotated models and find annotations for non-annotated model elements.

**Conclusions:**

Semantic propagation and model alignment are included in the open-source library semanticSBML, available on sourceforge. Online services for model alignment and for annotation prediction can be used at http://www.semanticsbml.org.

## Background

Systems biologists aim to understand the dynamic behaviour of cellular pathways with the help of quantitative models. To construct large-scale models flexibly from existing parts, models need to be retrieved, compared, and combined. For this purpose, modellers need to find and match equivalent model elements, for instance, variables describing identical metabolites. In the future, such alignments should be automated or, at least, sensible suggestions should be provided by software.

A simple way to match model elements is by comparing their names or identifiers as they appear in the model's code. Greedy alignments and model combination based on element names have been used in [1-4], but these approaches may fail if models stem from different sources and therefore use different naming schemes. A safer and more general approach is to compare model elements by the biological objects or concepts they stand for. Semantic annotations, for instance the MIRIAM-compliant annotations [5] used in the Systems Biology Markup Language (SBML) [6], provide a qualified naming scheme by relating model elements to entries in public web resources or ontologies. Knowledge from these ontologies can be used to compare alike biological objects and to define quantitative similarity scores between them [7,8].

Obviously, such comparisons will fail if annotations are missing. However, there are also algorithms that compare biological networks by their structures and can therefore handle missing annotations. Since the comparison of graph structures is computationally hard [9], these algorithms either yield approximative results [10] or are restricted to models having a simple structure like a path [11,12] or a tree [13,14]. The initial comparison of network nodes, which is later refined using structural information, also varies from approach to approach. While some of them use plain node labels, others compare nodes by chemical structures [10,15] or semantic information like EC numbers [16] or Gene Ontology terms [17]. With this information, they can either refine the alignments [18] or speed up the computations by reducing the search space [19]. A recent review on these and similar works can be found in [20].

In this article, we propose new heuristics for aligning network models with missing annotations. The basic idea is simple: a reaction, for instance in an SBML model, refers to its substrates and products. If two reactions are not annotated but their reactants are, we can trace the reactants, evaluate their annotations, and use this information for comparing the reactions. This logic can be applied whenever model elements show cross-references. Instead of collecting and combining this information step by step for each element, we developed a method to propagate all semantic information simultaneously across the network. Starting from an original *direct *similarity score, which compares elements only by their own annotations, we obtain a new *inferred *similarity score that incorporates information obtained from other elements.

Two applications of semantic propagation are presented in this article. The first one is the improved alignment of systems biology models. The second one is a method for predicting missing annotations in a model by aligning it to fully annotated network models. Our present implementation, which is a part of the tool semanticSBML [21], works for SBML models and for specific similarity measures [8]. However, semantic propagation is a general approach that applies to a wide range of network models and similarity scores and that can be combined with different model matching algorithms, including the ones mentioned above.

## Results

### Algorithm

We have developed methods for aligning partially annotated biological networks and implemented them for SBML models with MIRIAM-compliant annotations. A model alignment is based on two kinds of knowledge: similarities between model elements, which are computed from semantic annotations, and references between elements within each model (called here the "network structure").

**Figure 1:**
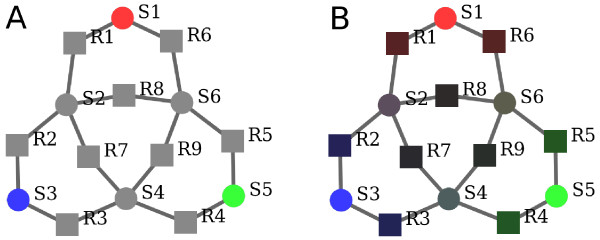
**Propagation of colour features**. Feature propagation in a small example network (circles: compounds; squares: reactions). A: Network nodes carry feature vectors *v *= (*r*, *g*, *b*)^T ^shown by RGB colours. Feature vectors for non-annotated elements are unknown and set to zero by definition (nodes shown in grey). B: After feature propagation, all nodes have distinct feature vectors, i.e. colours. Matching nodes by their colours would now allow to self-align the entire graph unambiguously.

The alignment of two models is computed in two steps. In a first step, semantic information is propagated across both network structures. This ensures that all elements, even elements lacking annotations, obtain semantic information, which makes them comparable between models. This claim of improved comparability is illustrated with an example of a propagation of "colour information" shown in Figure 1. Using the network structure, we refine the *direct *similarity *σ *and obtain an *inferred *similarity measure *ψ*, which can be nonzero even if one of the two compared elements does not carry any annotations.

In the second step, the actual model alignment, elements are matched between two or more models according to their inferred similarities. We use a greedy heuristic that arranges similar elements into tuples, supposed to be matched: all elements in a tuple have the same type (e.g. reaction), stem from different models, and each element is part of one tuple (possibly of size 1). Elements within a tuple have to show a high similarity, while elements in different tuples are supposed to be dissimilar.

#### Feature propagation

The semantic propagation can be carried out in two ways, as feature propagation (FP) or as similarity propagation (SP). For feature propagation, each element has to be associated with a feature vector. The components of this vector (usually numbers between 0 and 1) describe how closely the element resembles certain biological concepts. A model species describing a phosphorylated MAP kinase, for example, could carry annotations referring to UniProt entry P28482 (MAPK1) and KEGG Compound entry C00562 (Phosphoprotein). The feature vector corresponding to this species would contain mostly zeros except for the two entries specifying the relation to these web resource entries. Two elements are similar if their feature vectors point to similar directions, i.e. if they are related to similar biological concepts like a phosphorylated and a non-phosphorylated MAP kinase. The size of the feature vector depends on the number of biological concepts considered, e.g. the entries of all web resources being referred to in BioModels Database [22].

In feature propagation elements inherit semantic information from their neighbour nodes, which might contribute to the definition of the elements' identity. The inferred feature vectors do therefore not only characterise the model element itself, but also its surrounding elements in the network. In the case of a reaction element, they do not only describe the reaction, but also its reactants, enzyme, or regulators. The transition from the original to the inferred feature vectors is shown by an abstract example in Figure 1A. In this example the colours red, green, and blue correspond to the features in a colour vector of size three, which encode for semantic information. The propagation of this information (Figure 1B) leads to mixed colours in the center of the network, which give each node its own identity and allow for a unique self-alignment of the network. The mathematical details of feature propagation are explained in the Methods section.

#### Similarity propagation

**Figure 2:**
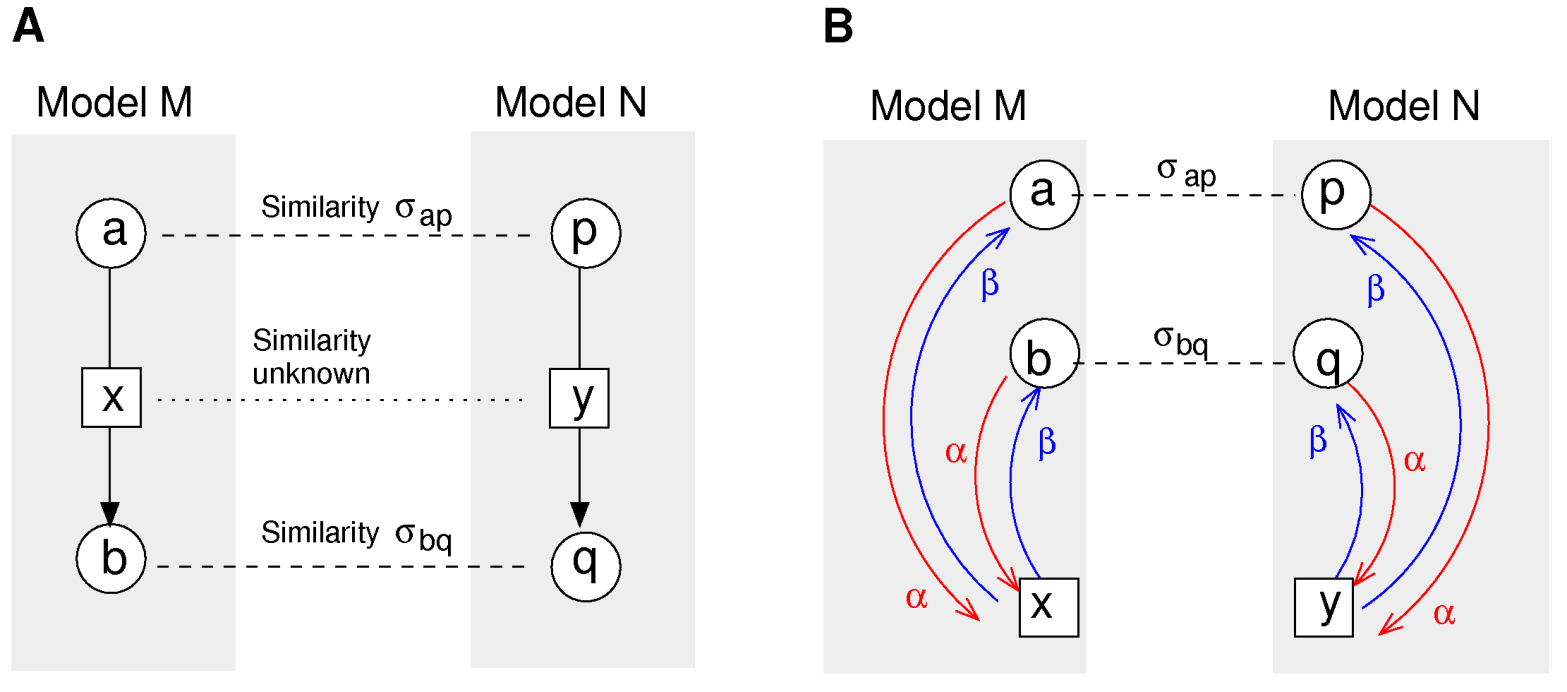
**Similarity propagation**. A: Alignment of two models describing the same biochemical reaction (circles: compounds; squares: reactions). The reactants a and p have a known similarity *σ_ap _*(dashed line; accordingly for products b and q), while the similarity between the reactions x and y is initially unknown. To determine how well the reactions match (dotted line), we compute the inferred similarities *ψ*^sp^. B: Propagation graph. Red arrows show how information is propagated from species to reactions (a potential propagation back is shown in blue). The similarity between the reactions x and y is supported by two paths (x ← a ↔ p → y and x ← b ↔ q → y). The respective terms *α*^2^*σ_ap _*and *α*^2^*σ_bq _*yield the inferred similarity $\psi_{xy}^{\text{sp}}=\alpha^{2}\left(\sigma_{ap}+\sigma_{bq}\right)$.

The second approach, similarity propagation, is computationally harder, but also applies to similarity measures that are not based on feature vectors. In contrast to FP, we do not consider single model elements, but pairs of elements from different models to be compared, e.g. the reactions *x *and *y *from Figure 2A. We require that, if two such elements refer to other elements that share a high similarity, this will increase their own similarity (Figure 2B). Our method determines these similarities in a self-consistent manner and uses a propagation process similar to feature propagation. In contrast to feature propagation the process is not carried out on the reaction network, but on an element pair graph. In this graph each node represents a pair of elements, one from each model. Two nodes are connected if the corresponding elements are connected in the reaction networks. The mathematical details are explained in the Methods section and a mathematical relation between feature and similarity propagation is discussed in additional file 1.

#### Annotation prediction

Model alignments can be used to complete missing annotations in a model. To do so, one may align the model to a large, annotated map of all physiological pathways and copy annotations from elements in the map to the model elements they have been aligned to. Even if not all of these annotations are correct, they may still be presented to users as suggestions during manual model annotation.

Our semanticSBML web page for model annotation (http://semanticsbml.org) provides this functionality and uses BioModels Database as a replacement for the large, annotated pathway map. The idea behind the annotation prediction is to apply feature propagation to a sparsely annotated model and all BioModels. Afterwards, the similarities between non-annotated model elements and all database elements are calculated. Finally, the annotations from the most similar elements contained in the database are presented to the user as suggestions for new annotations. Details of this procedure are described in the Methods section.

### Testing

#### Self-alignment of a linear chain

**Figure 3:**
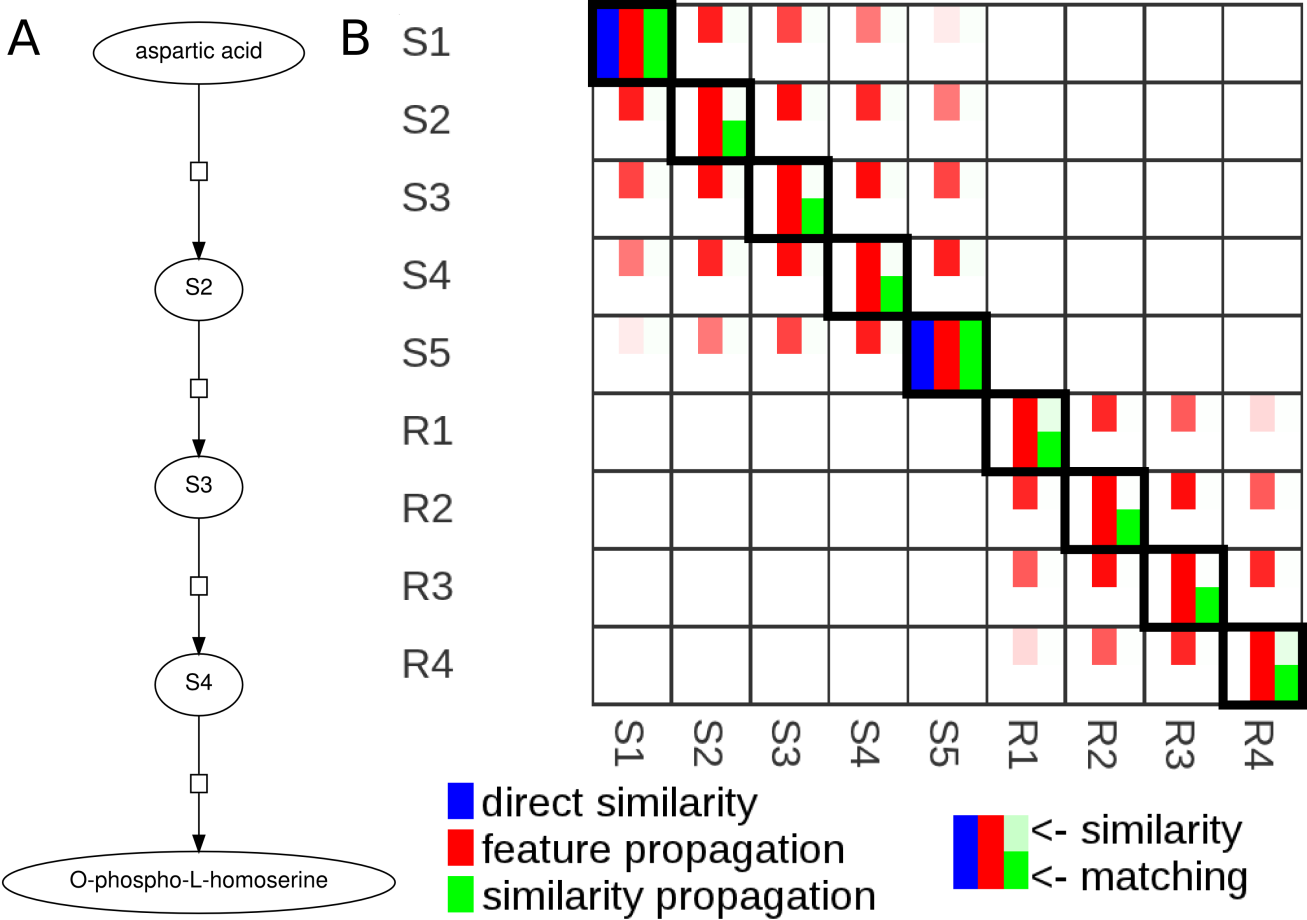
**Alignments of computational models**. A: Linear pathway; only the first and the last species are annotated. B: Self-alignment of the linear pathway. Depicted are the pairwise element similarities according to the three different similarity measures. Each table entry contains six numerical values. The entries in the upper line denote the direct similarity *σ *(blue), the similarity *ψ*^fp ^obtained by feature propagation (red), and the similarity *ψ*^sp ^from similarity propagation (green). Values between 0 and 1 are shown by colour intensities. Similarities between different types of elements (e.g. species and reactions) were set to 0, while *ψ*^sp ^values larger than 1 were set to 1. The lower three entries show the element matching obtained from these similarities. Thick boxes indicate the correct matching.

To illustrate how semantic propagation can improve model alignment, let us consider a linear metabolic pathway in which only the first and the last species are annotated (see Figure 3). If we align two copies of this pathway based on direct similarities, only these two elements can be matched. With the help of feature propagation or similarity propagation, all reactions and metabolites are matched correctly.

#### Alignment between MAP kinase pathways

**Figure 4:**
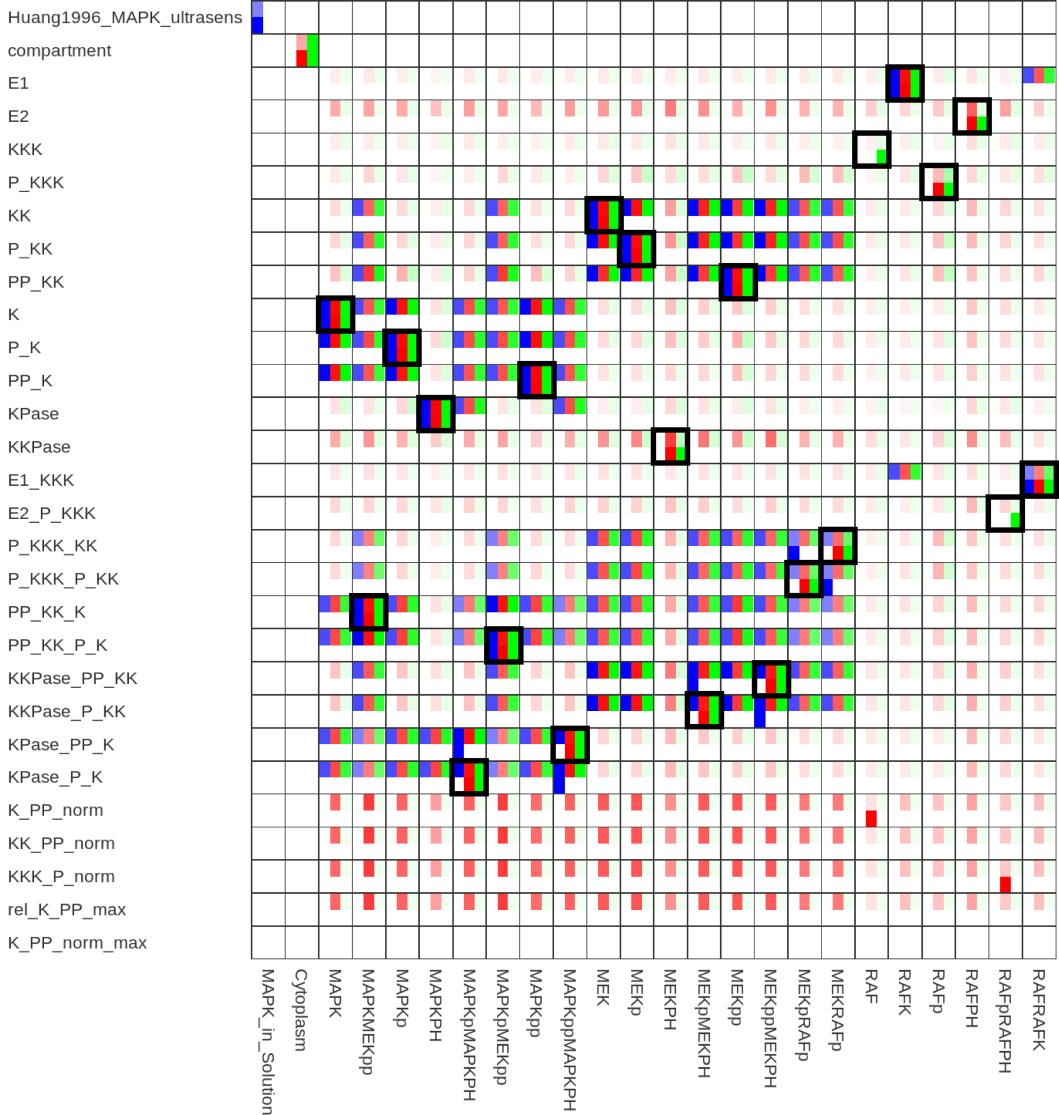
**Alignment of MAP kinase cascades**. Alignment of BioModels 9 [23] and 11 [24] as shown in Figure 4. The two alignments based on inferred similarities contain previously unmatched pairs (E2, RAFPH), (P_KKK, RAFp), and (KKPase, MEKPH) and some previously false matches are swapped. These incorrect matches resulted from the fact that elements represent proteins in different phosphorylation states but carry the same annotations. The inferred similarity measures differ in two details: the matching of RAF and RAFpRAFPH, which is correctly predicted from similarity propagation and the matching of Reaction19 and Reaction25, which is correctly obtained from feature propagation (data not shown).

As a real-world example, we aligned two well-annotated signal transduction models, model 9 [23] and model 11 [24] from BioModels Database. These models contain elements that represent proteins in different phosphorylation states but carry identical annotations. An alignment based on direct similarities would not be able to distinguish between these different states, but feature propagation and similarity propagation both manage to improve the results (see Figure 4).

**Figure 5:**
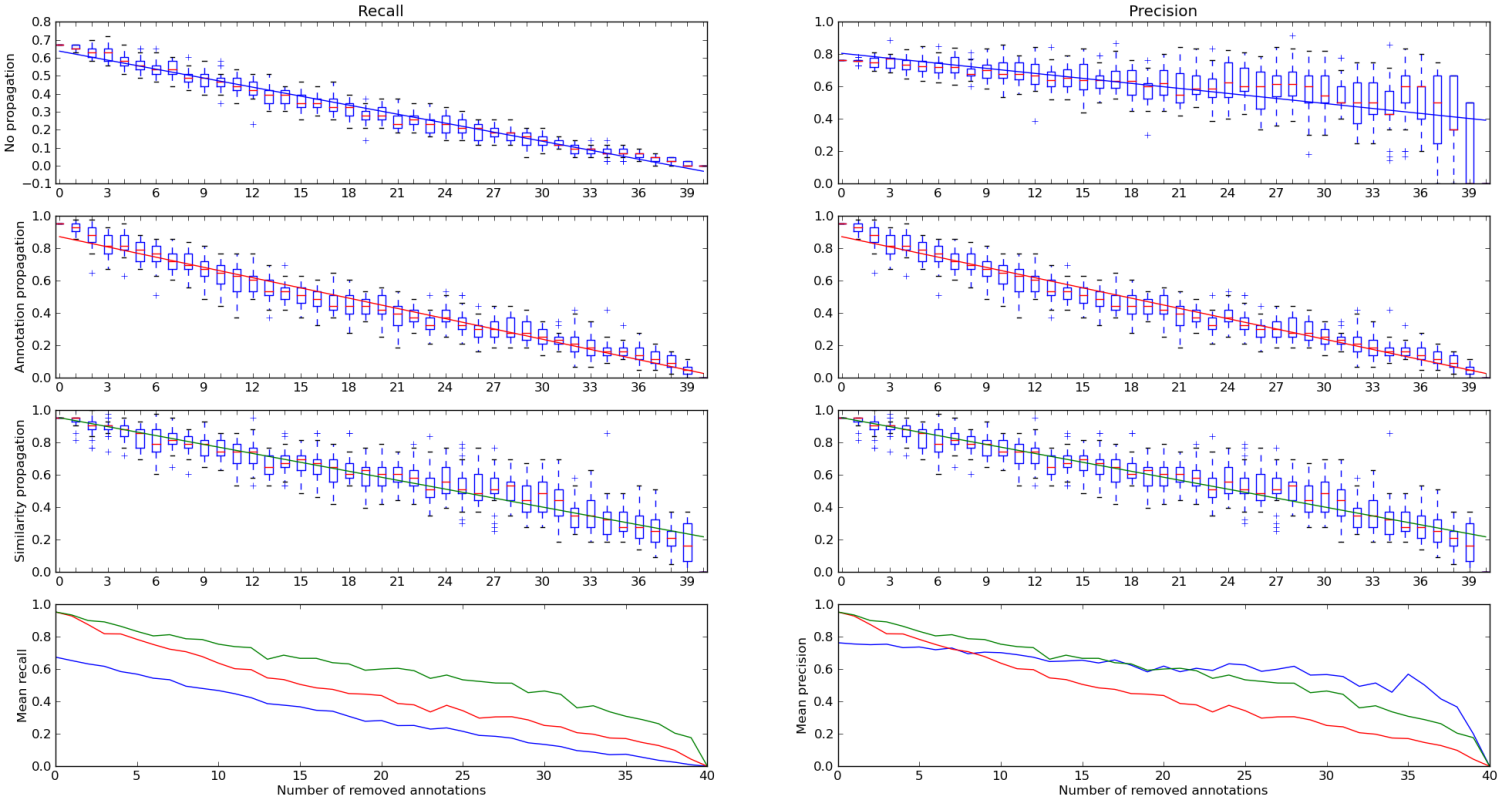
**Assessing the quality of model alignments**. The quality of model alignments increases with the fraction of annotated model elements. Alignments between BioModel 9 and BioModel 11 (see Figure 4) were compared to a manually chosen, correct alignment and scored by their recall (left boxes) and precision (right boxes). When annotations are randomly removed, recall and precision (y-axis) decrease with the fraction of removed annotations (x-axis). Boxes in different rows show results from alignments with direct similarities, FP similarities, and SP similarities, as well as a comparison of their three mean values. The straight lines in the three topmost boxes show trends and stem from a linear regression.

In practice, models are often less carefully annotated than these example models and the quality of a model alignment will depend on the fraction of annotated elements. To study this in detail, we considered the same two models, removed some of the annotations in BioModel 9 randomly, aligned the models, and scored the quality of the alignments by precision and recall with respect to a manually chosen alignment. This procedure was repeated 25 times for each number of removed annotations. Figure 5 shows the results: without semantic propagation, both precision and recall increase almost linearly with the number of annotations present. This is not surprising because the only possible reason for alignment errors are ambiguities caused by elements carrying identical annotations. If we use feature or similarity propagation, the quality increases more rapidly. As expected, the mean recall - i.e. the fraction of correct matches recovered - is improved because previously incomparable pairs can now be matched. The precision - counting how many predicted matches are actually correct - depends on how many elements are annotated. If many annotations have to be guessed, both inferred similarity scores yield a lower precision than the direct similarity. Nevertheless, with a higher number of annotations present, both methods can surpass the simple matching. SP outperforms FP for both precision and recall, but its computational effort is also much higher. Similar tests with different models lead to similar results (data not shown).

Despite the indifference of the results with respect to the models which are compared, the quality of the results varied in between examples in which only species or reaction annotations had been removed. The more detailed the annotations in a specific subset of model elements was, the more dramatic the decline in matching quality has been after their removal. For a deeper analysis of the matching quality after the removal of either species or reaction annotations from BioModel 9, the reader is referred to additional file 1.

#### Automatic suggestion of element annotations

In order to test the quality of our annotation prediction heuristic, we applied it to BioModel 61, a well-studied model of glycolysis [25]. For our first evaluation, we removed all annotations from the model and tried to re-predict them via the web interface. The test confirms that the predictions are not perfect, but the top 10 results usually contain relevant annotations. In general, the predictions become better as the fraction of correctly annotated model elements increases and as we scan more of the top results for the correct annotation.

**Figure 6:**
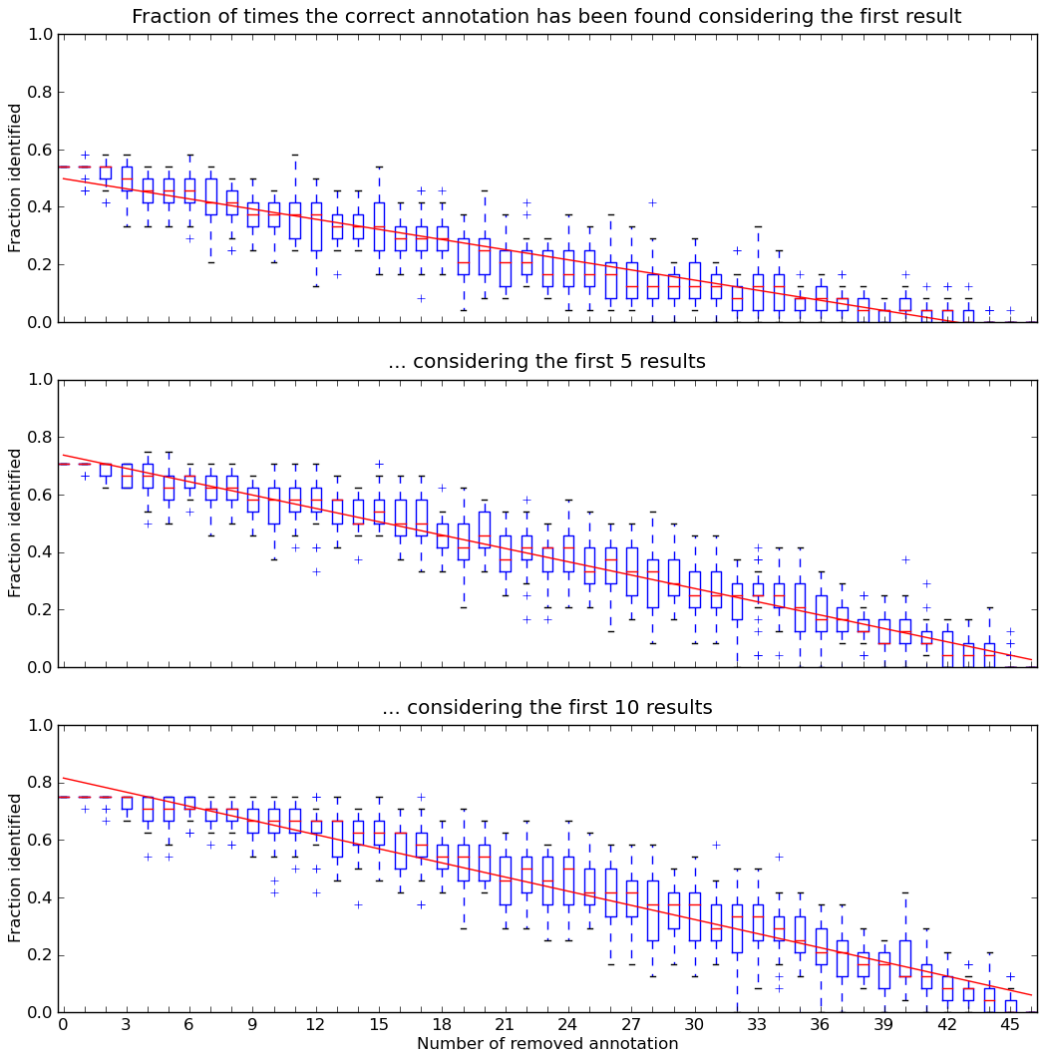
**Evaluation of the annotation prediction in BioModel 61**. Probability to find a correct annotation within in the first *n *predicted annotations (y-axis), for varying numbers of annotations present in the model (x-axis). Boxes show the cases *n *= 1, *n *= 5, and *n *= 10. Trends in the data are shown by straight lines.

To study this systematically, we randomly removed annotations from some of the elements, predicted new annotations for them, and checked if the correct annotation appeared in the top results. As shown in Figure 6, a significant number of annotations were predicted correctly using our propagation schemes. The results suggest that the probability of predicting an annotation correctly increases linearly with the number of annotations present in the model. Considering more of the topmost results did not strongly increase the quality of the prediction. This shows that correct annotations, if they can be predicted at all, will rank relatively high in the results.

### Implementation

We have implemented both propagation methods in Python. The source code of this implementation is included in our library semanticSBML, which is freely available (GPL 3) from SourceForge (http://sourceforge.net/projects/semanticsbml/) and can be used to e.g. annotate and merge SBML models.

Furthermore, we have included the semantic propagation methods into our web tool semanticSBML http://www.semanticsbml.org. First, this web tool allows users to visually compare the results of the three methods to compare model elements (direct similarity, feature propagation, and similarity propagation). Second, for model merging, all three methods can be used to suggest an initial matching of model elements. Third, for model annotation, new element annotations based on propagated features from BioModels will be suggested after the "Predict annotations" button has been clicked.

## Discussion

Annotating the elements of systems biology models is laborious. Interactive software can facilitate this work by keyword searches and by proposing annotations based on element names. Our software semanticSBML [21,26] helps modellers to annotate and combine SBML models based on MIRIAM-compliant annotations [5]. In the present work, we addressed an important open issue, the alignment of models with missing annotations.

As the examples have shown, element matchings based on our inferred similarity measures perform well in practice. In our tests, semantic propagation increased the recall of correct element pairs, but at the price of a lower precision if less than half of the elements were annotated. Our two approaches, feature and similarity propagation, differ slightly in their quality, but also in their computational costs. While similarity propagation tends to yield better results, the computational effort is much higher than in feature propagation $(O\left(|M{|}^{3}\cdot |N{|}^{3}\right)$ instead of $O\left(|M{|}^{3}+|N{|}^{3}\right)$, where |*M*| and |*N*| denote the numbers of elements in the two models).

We furthermore showed how new annotations for model elements can be suggested based on the annotations already present in a model and an annotated pathway map. For our predictions, we have selected the BioModels Database as a resource for highly curated annotation data. Analyses using other resources (see additional file 1 for details) show no loss in prediction quality and we plan to extend our background pathway map by using various other pathway resources in the future.

The details on how information is transfered during semantic propagation depends on the choice of propagation weights. In our tests, changing their values within reasonable ranges had little influence on the model alignment. In any case, the scaling factor *λ *(see Methods) allowed to avoid an overly strong propagation, which might lead to spurious similarities. As soon as a sufficient number of predefined model matchings will be available as a gold standard, the propagation weights may be further improved by machine learning.

## Conclusions

Compared to existing approaches, our methods for model alignment have three advantages. First, by processing semantic annotations instead of *ad-hoc *node labels, we can compare models from different sources and compare elements with similar, yet different annotations. Second, considering the network structure allows us to distinguish between model elements carrying identical annotations, e.g. proteins in different phosphorylation states. Third, our similarity measures can be combined with various structure-based model comparison algorithms, e.g. [13]. Although our approach has only been implemented for SBML models, it can be extended to any computational models that include structural information and semantic annotations.

## Methods

### Semantic similarity

The biological meaning of a model element can be described with the help of annotations, which point to entries in different web resources. Since different web resources overlap in their content (e.g. ChEBI [27] and KEGG Compound [28]), entries from different resources can have the same biological meaning, i.e. describe the same *biological concept*, e.g. as ChEBI entry CHEBI:17925 and KEGG Compound entry C00267 both describing *β*-D-glucose. Given a list of biological concepts, we can describe the meaning of a model element *x*, e.g. a species, a reaction, or a compartment, by a *feature vector ***v***_x_*. This vector contains non-zero values **v**_*x*,*i *_only for those biological concepts that appear in the element's annotations. If, for example, element *x *has been annotated using the aforementioned ChEBI entry CHEBI:17925 or the KEGG Compound entry C00267 (and assuming that the *j^th ^*biological concept is describing *β*-D-glucose), the value of **v**_*x*,*i *_will be larger than zero. Similarities *σ *between two model elements *x *and *y *can be computed by taking the cosine of the angle between their feature vectors [29]

$$\sigma_{xy}^{\text{e}}=\frac{v_{x}^{\text{T}}v_{y}}{\sqrt{v_{x}^{\text{T}}v_{x}}\sqrt{v_{y}^{\text{T}}v_{y}}},$$

setting $\sigma_{xy}^{\text{e}}=0$ if either $v_{x}=\vec{0}$ or $v_{y}=\vec{0}$. This similarity measure assumes that different biological concepts are logically independent and completely dissimilar: the basis vectors, each representing one concept, are orthogonal and therefore share similarities of 0. To allow for potential similarities between them, e.g. to allow for a non-zero similarity between *β*-D-glucose and D-glucose, we may predefine similarities *S_ij _*between the biological concepts and replace the scalar product by a quadratic form based on the matrix *S*. The new similarity measure reads

(1) $$\sigma_{xy}=\frac{v_{x}^{\text{T}}Sv_{y}}{\sqrt{v_{x}^{\text{T}}Sv_{x}}\sqrt{v_{y}^{\text{T}}Sv_{y}}}$$

with *σ_xy _*= 0 whenever one of the elements is not annotated [30]. For ways to derive such a matrix *S*, the reader is referred to [8].

### Propagation of semantic information

To compare non-annotated model elements by their biological meaning (e.g. the reactions in Figure 2A), we have developed two alternative propagation schemes. In *feature propagation *(FP) elements receive information about semantic annotations from their neighbours, while in *similarity propagation *element pairs receive information about pairwise similarities between neighbouring elements. In both methods information is propagated along the references between model elements (Figure 2B). The strength of information transfer is determined by a |*M*| × |*M*| *propagation matrix ρ*^M ^where |*M*| is the number of elements in model M. The sparse structure of this matrix reflects the connections between model elements, and its positive, real-valued entries $\rho_{xa}^{\text{M}}$ determine the strength of the information transfer between the different types of elements. For instance, we may choose the values $\rho_{xa}^{\text{M}}=\alpha$ and $\rho_{ax}^{\text{M}}=\beta$ whenever a reaction *x *refers to a species *a *as its reactant or product, and $\rho_{xa}^{\text{M}}=0$ if *a *does not appear in reaction *x*. The parameter *α *controls the information transfer from species to reactions, while the parameter *β *controls information transfer in the opposite direction (Figure 2B). To prevent semantic propagation between unrelated reactions, we stop the propagation at cofactors, which might be highly connected hubs in the network structure. Therefore, we set $\rho_{ax}^{\text{M}}=\rho_{xa}^{\text{M}}=0$ whenever the annotations on a species *a *suggest that it is a cofactor (see additional file 1 for details).

**Feature propagation **applies to all similarity scores that are computed from feature vectors. For each *direct *feature vector **v***_x_*, we define a new *inferred *feature vector **w***_x_*, which is supposed to resemble the inferred feature vectors of the neighbouring elements in the network (see Figure 1B). It is defined by the equation

(2) $$w_{x}=v_{x}+\lambda \sum_{a\in M}\rho_{xa}^{\text{M}}w_{a},$$

with *λ *being a scaling factor which is determined below. If an element *x *does not receive any information from other elements (i.e. $\forall_{a\in M:}\rho_{xa}^{\text{M}}=0$), its inferred and its direct feature vector are identical, i.e. **w***_x _*= **v***_x_*. In all other cases, we add the inferred feature vectors of all referenced elements, multiplied by their propagation weights *ρ_xa _*and *λ*. To compute **w***_x _*from Eq. (2), we consider each feature *i *separately. We collect the *i*^th ^components of all feature vectors **v***_x _*in a column vector v_*,*i *_and the *i*^th ^components of all vectors **w***_x _*in a vector w_*,*i*_. With the |*M*| × |*M*| matrix $R=\left(\rho_{xa}^{\text{M}}\right)$, each component of the vectors in Eq. (2) can be written as

(3) $$\begin{array}{llll} w_{*,i} & =v_{*,i}+\lambda R\;w_{*,i}\; & & \;\\ & ={\left(I-\lambda R\right)}^{-1}v_{*,i}\; & & \;\\ & \; & \end{array}$$

After choosing a scaling factor *λ *that is smaller than the inverse of the biggest absolute eigenvalue *r *of *R *$\left(\text{e}.\text{g}.\lambda =\frac{1}{2r}\right)$, we can replace the matrix inverse in Eq. (3) by

(4) $$w_{*,i}=\overset{\infty }{\underset{k=0}{\sum }}{\left(\lambda R\right)}^{k}v_{*,i}.$$

The series expansion shows that the inferred feature vectors can be obtained by summing up a number of terms, describing how the direct features are propagated step by step through the network, e.g. from compartments to molecular species, from reactants to reactions, and so on. If information is propagated only in one direction on an acyclic graph, these contributions can be computed successively and the infinite series contains only a finite number of non-zero terms. Moreover, the series expansion shows that the inferred feature vectors are non-negative as long as the direct feature vectors and the propagation weights are nonnegative. By collecting the vectors v_*,*i *_and w_*,*i *_in matrices *V *and *W*, the equations (4) for all elements *i *can be joined into a single matrix equation

(5) $$W={\left(I-\lambda R\right)}^{-1}V.$$

In analogy to Eq. (1), the element similarity with inferred features reads

(6) $$\psi_{xy}^{\text{fp}}=\frac{w_{x}^{\text{T}}Sw_{y}}{\sqrt{w_{x}^{\text{T}}Sw_{x}}\sqrt{w_{y}^{\text{T}}Sw_{y}}}.$$

#### Similarity propagation

Our second scheme, similarity propagation (SP), works for any similarity score. Given the direct similarities *σ_xy _*(from Eq. (1) or elsewhere) between elements *x *in model *M *and elements *y *in model *N*, we define the inferred similarities by

(7) $$\psi_{xy}^{\text{sp}}=\sigma_{xy}+\lambda \underset{a\in M,p\in N}{\sum }\rho_{xa}^{\text{M}}\;\psi_{ap}^{\text{sp}}\rho_{yp}^{\text{N}}.$$

The idea is analogous to Eq. (2): the inferred similarity $\psi_{xy}^{\text{sp}}$ represents the direct similarity *σ_xy _*plus the weighted inferred similarities between all elements *a *and *p *to which *x *and *y *refer. To solve Eq. (7), we merge double subscripts (as indicated by brackets) and rewrite it as

$$\psi_{xy}^{\text{sp}}=\sigma_{\left(xy\right)}+\lambda \underset{\left(ap\right)}{\sum }Q_{\left(xa\right)\left(yp\right)}\psi_{\left(ap\right)}^{\text{sp}},$$

where $Q_{\left(xa\right)\left(yp\right)}=\rho_{xa}^{\text{M}}\rho_{yp}^{\text{N}}$. Using the vectors $\psi^{\text{sp}}=\left(\psi_{\left(xa\right)}^{\text{sp}}\right)$ and *σ *= (*σ*_(*xa*)_) and the matrix *Q *= (*Q*_(*xa*)(*yp*)_), the solution can again be written as

(8) $$\psi^{\text{sp}}={\left(I-\lambda Q\right)}^{-1}\sigma .$$

Like in Eq. (4), if *σ *is non-negative, *λ *can be chosen such that *ψ*^sp ^is also non-negative. As an example, explicit calculations of both similarity measures for a partially annotated model of the phosphoglucoisomerase reaction are shown in section 5 in additional file 1.

#### Analogy to diffusion processes

Feature propagation resembles a diffusion process on the reaction network. Unlike diffusion in the strict sense, our process is not symmetric and does not conserve mass. Methods with a similar background idea have already been successfully applied to assign protein functions in protein-protein interaction networks [31,32].

**Figure 7:**
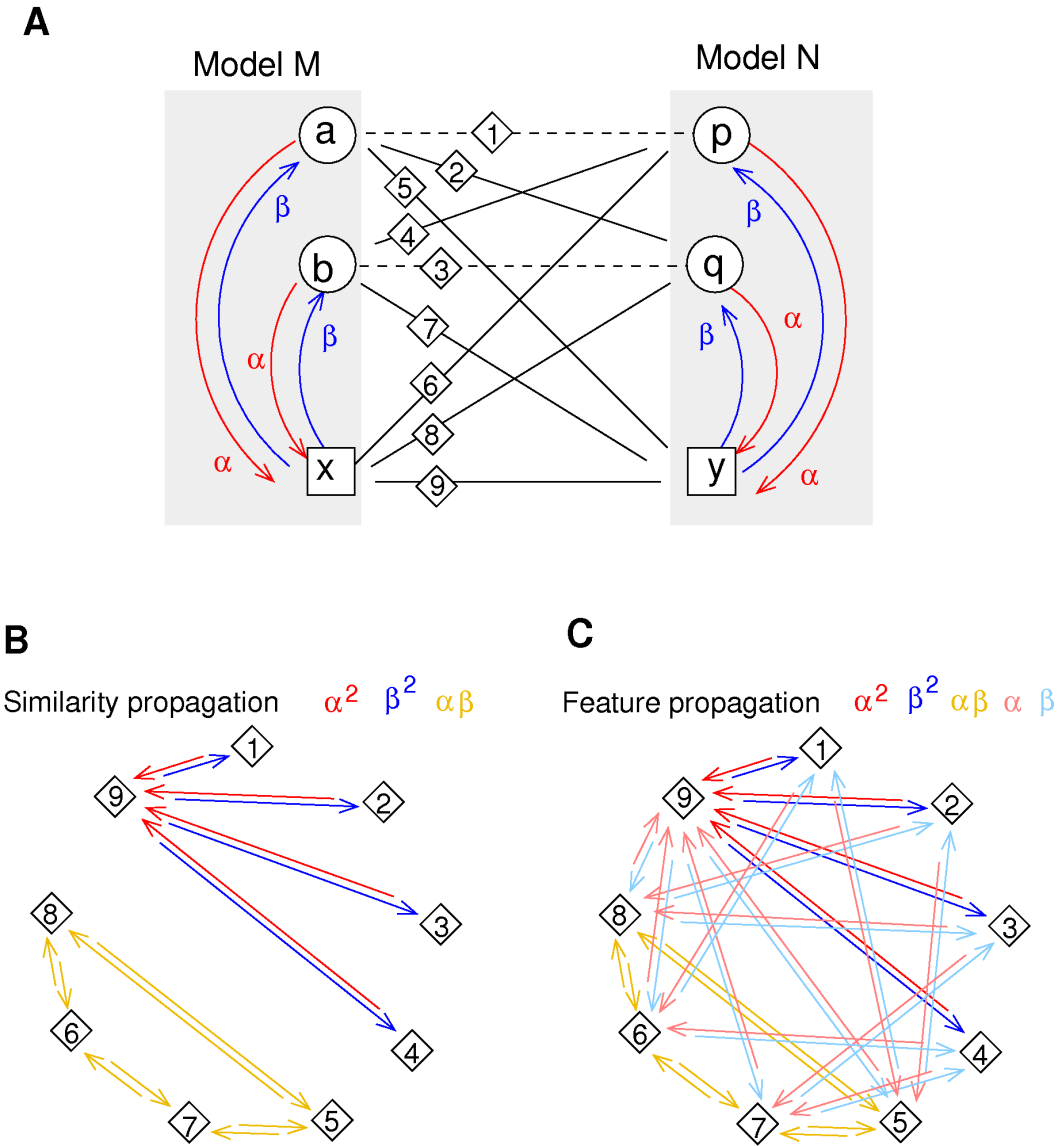
**Similarity propagation resembles diffusion of a graph**. A: Propagation graph from Figure 2, redrawn with labels for element pairs (diamonds). B: Similarity propagation on a *pair propagation graph*. Nodes in the graph correspond to element pairs in the original networks. Edges indicate that information can be propagated simultaneously for both elements in a pair. For instance, similarity information can be propagated from the pair (a,p) to the pair (x,y), resulting in the edge 1 → 9 with a weight *α *· *α*. C: Feature propagation entails a similar process with more paths for propagation. For instance, similarity information could now be propagated from the pair (a,y) to the pair (x,y), resulting in the edge 5 → 9 with weight *α *· 1.

Likewise, similarity propagation can be seen as a diffusion-like process on a *pair propagation graph *in which each node corresponds to a pair (*x*, *y*) of elements from the two models. An example is shown in Figure 7. An edge from node (*a*, *p*) to node (*x*, *y*) in this graph $\left(\text{with weight}\;\rho_{xa}^{\text{M}}\rho_{yp}^{\text{N}}\right)$ means that semantic information is propagated from *a *to *x *$\left(\text{with weight }\rho_{xa}^{\text{M}}\right)$ and from *p *to *y *$\left(\text{with weight }\rho_{yp}^{\text{M}}\right)$. The matrix *Q*_(*xa*)(*yp*) _serves as a non-symmetric, non-mass-conserving diffusion matrix for this graph. To describe semantic propagation as a temporal process on this graph, we may introduce a production term *σ *and a linear degradation term. We then obtain the equation

$$\frac{\text{d}}{\text{d}t}\psi^{\text{sp}}=\sigma +Q\;\psi^{\text{sp}}-\psi^{\text{sp}},$$

whose stationary distribution is given by Eq. (8).

The structure of the pair propagation graph determines how information about element similarities can spread during propagation. For instance, the graph in Figure 7B shows that an initial matching between species (edges 1, 2, 4, and 5) or between reactions (edge 9) cannot lead to a matching between species and reactions (edges 3, 6, 7, and 8) because they appear in separate subgraphs.

### Model alignment

A main task in model merging is to detect and combine corresponding elements between models. In such a model alignment the element matching has to be non-ambiguous (an element cannot be matched to several elements in another model) and transitive (if *x*, *y*, and *z *are elements from three different models and if the pairs (*x*,*y*) and (*y*,*z*) are matched, the pair (*x*,*z*) is matched automatically).

Given two models *M *and *N *with elements *x *and *y*, a model alignment can be defined as a set P={(x,y),(a,p),(b,q),...} of disjoint element pairs. If more than two models are aligned, we may also obtain triples or larger tuples of elements. All remaining single elements are also treated as tuples. To find a good alignment P, we use precalculated element similarities *ψ_xy _*and evaluate the score function

(9) $$f=\underset{\left(xy\right)\in P}{\sum }\psi_{xy}.$$

Instead of maximising the score exactly (which would be computationally hard), we employ a greedy algorithm: first, it chooses the element pair with the highest *ψ *value. If this value is positive, the two elements are matched and the pair is removed from the list. Then, we continue to match the element pairs with the highest remaining *ψ *value until no positive *ψ *values are left. If this leads to inconsistencies (i.e, if one of the two elements already has a different matching partner in the other model), the next best matching pair is considered instead. In the end, the alignment score is given by the sum of all *ψ *values collected.

For aligning three or more models, we use the same matching score (for element pairs), but consider the transitivity constraint: Whenever we match two elements, we also match all their previously determined matching partners (and consider the respective mutual similarities in the score function).

### Finding element annotations based on model alignments

Semantic propagation can be used to suggest annotations for non-annotated model elements. For this purpose the considered partially annotated model is aligned to a fully annotated large pathway map. Afterwards, annotations are transferred from elements in the map to the corresponding, non-annotated elements in the model.

As a prerequisite, we collected many annotated model elements appearing in BioModels Database, which served as a replacement for the large, annotated pathway map, and calculated their propagated feature vectors. For each element (*x *∈ *M*) in each of the models, its propagated feature vector (**w***_x_*) is computed. For every annotation *i *on the element $\left(\forall_{i:v_{*,i}\neq 0}\right)$ we do the following. The corresponding feature is removed from the vector (**w**_*x*,*i *_= 0) and the pair of the feature and this propagated feature vector is added to the collection.

To find annotations for a non-annotated element of interest, we calculate its propagated feature vector within its model and check if there are similar vectors in our collection. Afterwards, the annotations associated with these vectors are presented to the user. The general idea is that given a model from the BioModels Database that lacks annotations for a single feature on a given element, this method would be able to predict annotations for this element with perfect accuracy.

For annotation prediction on the semanticSBML website, the feature propagation algorithm has been slightly modified. To reduce the number of annotations suggested, we set the propagation values *ρ *between reaction - reactant/product to $\frac{1}{2}$ and all others to 0. Moreover, information is propagated only to direct neighbours (i.e. the sum in Eq. (4) does not go to infinity but to 1).

## Authors' contributions

MS and WL developed the semantic propagation and wrote the paper. MS implemented the algorithms and analysed the data. MS, EK, and WL designed computational experiments. All authors read and approved the final manuscript.

## Supplementary Material

Additional file 1 **Appendix**. Text document containing details on the implementation, a further comparison of the methods, an additional numerical example, and further analyses. Click here for file
