# Supplementary material for: Propagating semantic information in biochemical network models
Source: BMC Bioinformatics. 2012 Jan 30;13:18. doi: 10.1186/1471-2105-13-18 (PMC3340317; doi:10.1186/1471-2105-13-18)
Supplement: Additional file 1 — Appendix. Text document containing details on the implementation, a further comparison of the methods, an additional numerical example, and further analyses. [file 1471-2105-13-18-S1.PDF]

Propagating semantic information in biochemical network models

Supplementary Appendix

Marvin Schulz, Edda Klipp, and Wolfram Liebermeister

Abstract

In this Supplementary Appendix, we first describe in detail which information can be propagated in models described in the Systems Biology Markup Language. We further show how the two propagation schemes discussed in the main text - feature propagation and similarity propagation - are mathematically related. Then, we discuss ways to normalise propagated similarities and explain how our scoring function for model alignments can be derived from probabilistic considerations. As an illustrative example of feature and similarity propagation, we explicitly calculate both similarity scores for two models of the phosphoglucoisomerase reaction. Finally, we take a deeper look into some of the analyses performed in the main text. We investigate the effects of cofactors on the alignments of linear chains, study how removing annotations only from species or only from reactions affects the prediction of annotations, compare alignments for models containing lumped elements, and check whether a different large pathway map affects the quality of the annotation prediction.

Contents

1 Implementation of semantic propagation for SBML models **2**

2 Mathematical differences between feature propagation and similarity propagation **3**

3 Scaling of similarity values **4**

4 Score function for model alignments **4**

5 Simple example: unimolecular reaction **5**
5.1 Feature propagation 5
5.2 Similarity propagation 7

6 Semantic propagation through cofactors **8**

7 Comparing alignment quality after removal of annotations from different types of nodes **9**

8 Alignment of models described to different detail **10**

9 Using KEGG as a reference database for annotation prediction **10**

1 Implementation of semantic propagation for SBML models

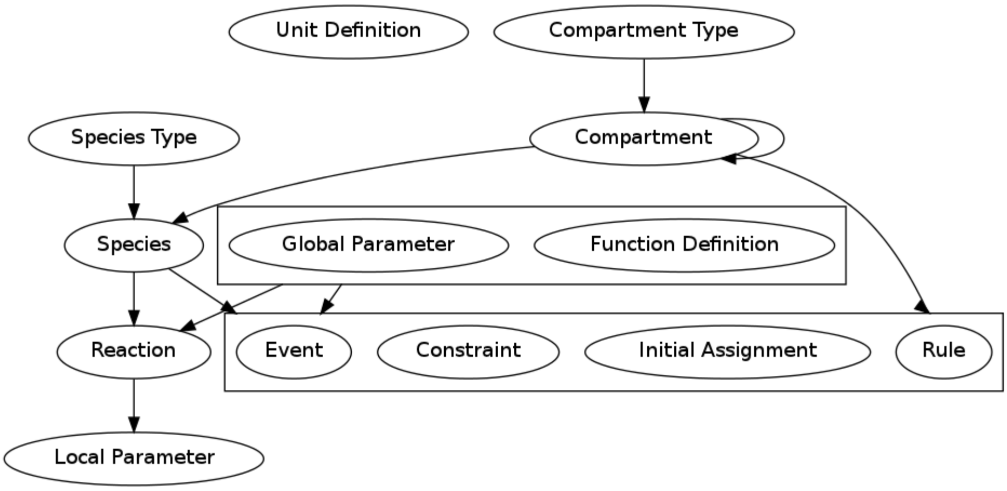

Figure S1: References between SBML elements. SBML elements can refer to other SBML elements in their definition: For instance, reactions refer to chemical species as their reactants or modifiers, chemical species can be localised in a cellular compartment, and compartments can be localised within other compartments. All possible references shown can be used to propagate semantic information. Arrows between element types are assigned propagation weights ρ and possibly other weights for propagation in the opposite direction. With unidirectional propagation arrows, information is propagated step by step from top to bottom.

The elements of an SBML model describe biochemical objects or processes (chemical species, reactions, cell compartments, events etc.) or mathematical objects (physical units, functions, equations). These elements can be linked by various kinds of relations: e.g. a reaction has several reactants and each of them is located in a certain compartment. Individual elements can carry MIRIAM-compliant annotations, which allow to compute semantic similarities between them. For non-annotated elements, a semantic similarity cannot be easily calculated because we have no direct evidence of their underlying biological concepts (e.g. being a certain metabolite, protein, or process). Nevertheless, we can infer hints on their identity from the annotations of the elements to which they are related. While the main text discusses only relations between species and reactions, also other relations in SBML can be used to propagate semantic information. An overview of element types and possible references between them is given in Figure S1.

Elements in SBML can refer to the IDs of other elements and thereby inherit those elements' identities. A good example for this transfer of semantic information are compartment types and species types. If the same species is supposed to be defined in various compartments, all the information these species elements have in common can be assigned to a species type element. Then, all the species elements can reference this species type and only have to be assigned the specific information that distinguishes them. Since information about the biological concept behind the species could be stored in the species type, it is clear that each individual species should inherit the features associated with its species type and its compartment. Another example are rules and events. These elements should inherit semantic information from the elements on which they act. For the relations shown in Supplementary Figure S1, information is sent along the reference arrows,

possibly in both directions, and each kind of reference (e.g. “reactant” or “modifier”) can have a different propagation weight.

For the practical applications described in the main article, we use two sets of propagation weights ρ: (i) for model merging, all weights are set to $\frac{1}{2}$; (ii) for annotation prediction, weights between reactions and their reactants or products are set to $\frac{1}{2}$ and all others to 0. These values have been chosen *ad hoc* and based on heuristic refinements. In the future, curated sets of model alignments may permit to obtain better values by machine learning.

2 Mathematical differences between feature propagation and similarity propagation

Feature propagation and similarity propagation are closely related. To see this, let us compare the results from both methods for a simple similarity score, the scalar product $\sigma_{ap} = \mathbf{v}_a^{\mathrm{M}} \cdot \mathbf{v}_p^{\mathrm{M}}$ between feature vectors, and for $\lambda = 1$.

1. The similarities obtained from similarity propagation, as depicted in Figure 7 in the article, read

$$\begin{aligned} \psi^{\mathrm{sp}}_{(ap)} &= \big((I - Q)^{-1} \operatorname{vec}(\mathbf{v}^{\mathrm{M}} \otimes \mathbf{v}^{\mathrm{N}})\big)_{(ap)} \\ &= \big(\big(\delta + Q + Q^2 + ...\big) \operatorname{vec}(\mathbf{v}^{\mathrm{M}} \otimes \mathbf{v}^{\mathrm{N}})\big)_{(ap)} \\ &= \mathbf{v}_a^{\mathrm{M}} \cdot \mathbf{v}_p^{\mathrm{N}} + \sum_{xy} \rho^{\mathrm{M}}_{ax} \, (\mathbf{v}_x^{\mathrm{M}} \cdot \mathbf{v}_y^{\mathrm{N}}) \, \rho^{\mathrm{M}}_{py} \\ &\quad + \sum_{xbyq} \rho^{\mathrm{M}}_{ax} \, \rho^{\mathrm{M}}_{xb} \, (\mathbf{v}_b^{\mathrm{M}} \cdot \mathbf{v}_q^{\mathrm{N}}) \, \rho^{\mathrm{M}}_{py} \, \rho^{\mathrm{M}}_{yq} + ... \end{aligned} \tag{S1}$$

   where a, b, and x denote elements from one model, p, q, and y denote elements from the other, $\otimes$ represents the Kronecker product, and vec vectorises a matrix by stacking all its columns into a single column. The first term in this series compares the two elements a and p directly by their explicit similarity, the second term compares all elements from which they directly receive information, the third term compares the elements that are one step further apart, and so on.

2. Using feature propagation, we can first determine the propagated feature vectors $\mathbf{w}_a$ and $\mathbf{w}_p$ of the model elements and then compute their scalar product. The resulting similarities read

$$\begin{aligned} \psi^{\mathrm{fp}}_{ap} &= \mathbf{w}_a \cdot \mathbf{w}_p \\ &= \left(\mathbf{v}_a^{\mathrm{M}} + \sum_x \rho^{\mathrm{M}}_{ax} \, \mathbf{v}_x^{\mathrm{M}} + \sum_{xb} \rho^{\mathrm{M}}_{ax} \, \rho^{\mathrm{M}}_{xb} \, \mathbf{v}_b^{\mathrm{M}} + ...\right) \\ &\quad \cdot \left(\mathbf{v}_p^{\mathrm{N}} + \sum_y \rho^{\mathrm{N}}_{py} \, \mathbf{v}_y^{\mathrm{N}} + \sum_{yq} \rho^{\mathrm{N}}_{py} \, \rho^{\mathrm{N}}_{yq} \, \mathbf{v}_q^{\mathrm{N}} + ...\right). \end{aligned}$$

   After expanding this expression, we obtain the same terms as in Eq. (S1), but also additional mixed terms. For instance, we obtain terms comparing a directly to all elements from which b inherits information. Some of these terms will vanish because they compare elements of different types (e.g. reactions and species), which are annotated using different ontologies.

The additional terms in feature propagation can be an advantage. For instance, they help to align models containing lumped reactions because the exact distance between two elements

(e.g. two metabolites separated either by several reactions or by one lumped reaction) will matter less than in similarity propagation. Furthermore, they allow to handle cases in which one model describes an enzyme as a chemical species, while the enzyme annotation is attached to the catalysed reaction in the other model.

3 Scaling of similarity values

Similarities are usually expressed on a scale between 0 and 1. In the FP score (Eq. (6) in the article), this is automatically ensured because the explicit feature vectors and the propagation weights, and therefore also the propagated features, are non-negative. In similarity propagation, in contrast, similarity scores between 0 and 1 can be obtained in different ways: First, if the explicit similarities and propagation weights are positive, the propagated similarities will also be positive. To avoid values larger than 1, we can either use 1 as a cut-off value or we normalise each row and column of the similarity matrix by the square root of its maximal values, thus replacing it by

$$\psi^{\text{sp}*}{}_{xy} = \frac{\psi^{\text{sp}}_{xy}}{(\sqrt{\max_{p \in N} \psi^{\text{sp}}_{xp}} + \varepsilon)(\sqrt{\max_{a \in M} \psi^{\text{sp}}_{ay}} + \varepsilon)}.$$

The small positive term ε is added only to avoid a division by zero. Finally, similarity propagation may be applied not to the original similarity scores $\sigma \in]0,1[$, but to their logit transform

$$\sigma' = \ln \frac{\sigma}{1-\sigma} \in \mathbb{R}. \tag{S2}$$

After similarity propagation, we obtain propagated transformed scores $\psi^{\text{sp}\prime}$ and may use the reverse transformation (i.e. a logistic transformation) to obtain inferred similarities

$$\psi^{\text{sp}} = \frac{e^{\psi^{\text{sp}\prime}}}{1 + e^{\psi^{\text{sp}\prime}}} \in]0,1[. \tag{S3}$$

The logistic transformation is a natural choice if we interpret the scores σ as probabilities. When assigning annotations to a model element, the annotator has a certain biological entity in mind, which we call the *intended biological concept* (IBC). The annotations should describe the IBC as precisely as possible, but they will often remain vague or even be incorrect. Before merging two model elements x and y, we need to decide whether their IBCs x^{I} and y^{I} are biochemically close enough to justify an element matching (or, for short, whether x and y are *matchable*: $x \equiv y$). Since we do not know the IBCs themselves, we cannot decide this with certainty, but have to incorporate evidence given by the annotations.

Therefore, we may regard the similarity score σ_{xy} as the probability that x and y are matchable, given all information contained in the annotations. In this case, the transformed score σ' will be the log odds ratio, and similarity propagation yields a sum of such log odds ratios. Using the logistic transformation Eqs. (S2) and (S3), our final similarity scores cannot exactly reach values of 0 or 1. This is not a mathematical artifact, but expresses the uncertainty whether annotations do completely and uniquely describe the IBCs.

4 Score function for model alignments

Our score function for model alignments – the summed similarities of all matched element pairs in Eq. (8) – can be justified by the same probabilistic interpretation. Let us assume that the untransformed similarity $\psi_{xy} \in]0,1[$ is the probability that two elements are matchable. For

scoring a model alignment, we now consider the logarithmic probability that all matched element pairs are matchable and that all non-matched element pairs are non-matchable. We obtain

$$
\begin{aligned}
\log\left(\prod_{(x,y)\in\mathcal{P}} p(x \equiv y) \prod_{(x,y)\notin\mathcal{P}} p(x \not\equiv y)\right) &\approx \sum_{(x,y)\in\mathcal{P}} \log\psi_{xy} + \sum_{(x,y)\notin\mathcal{P}} \log(1-\psi_{xy}) \\
&= \sum_{(x,y)\in\mathcal{P}} [\log\psi_{xy} - \log(1-\psi_{xy})] + \sum_{(x,y)} \log(1-\psi_{xy}) \\
&= \sum_{(x,y)\in\mathcal{P}} \left[\log\frac{\psi_{xy}}{1-\psi_{xy}}\right] + C = \sum_{(x,y)\in\mathcal{P}} \psi'_{xy} + C.
\end{aligned}
$$

As above, the values ψ' are logistically transformed similarities, while C is a constant.

5 Simple example: unimolecular reaction

For illustration, we consider simple models for a reaction in glycolysis, the phosphoglucoisomerase reaction, which converts glucose 6-phosphate into fructose 6-phosphate. The problem has the same structure as the one shown in Figure 2 A, with the propagation scheme shown in Figure 2 B.

5.1 Feature propagation

For feature propagation, we first determine the original feature vectors, summarised in a feature matrix V. Originally, the matrix V has as many columns as there are biological concepts described in various web resources:

V=

...	glucose	glucose 6-phosphate	fructose 6-phosphate	fructose 1,6-bisphosphate	...	
0	0	1	0	0	0	a
0	0	0	1	0	0	b
0	0	0	0	0	0	x

but we trim it to the only two available biological concepts. Since a is annotated as glucose 6-phosphate, b is annotated as fructose 6-phosphate, and x is not annotated, we end up with

$$V = \begin{pmatrix} 1 & 0 \\ 0 & 1 \\ 0 & 0 \end{pmatrix}.$$

Next, we calculate the inferred feature matrix W for each model (see Eq. (5)), which contains the inferred feature vectors

$$\begin{aligned} W &= \left(I - \lambda \begin{pmatrix} \rho_{aa} & \rho_{ab} & \rho_{ax} \\ \rho_{ba} & \rho_{bb} & \rho_{bx} \\ \rho_{xa} & \rho_{xb} & \rho_{xx} \end{pmatrix} \right)^{-1} V \\ &= \left(I - \lambda \begin{pmatrix} 0 & 0 & \beta \\ 0 & 0 & \beta \\ \alpha & \alpha & 0 \end{pmatrix} \right)^{-1} V. \end{aligned}$$

Matrix W reads

$$\begin{aligned} W &= \frac{1}{2\alpha\beta\lambda^2 - 1} \begin{pmatrix} \alpha\beta\lambda^2 - 1 & -\alpha\beta\lambda^2 & -\beta\lambda \\ -\alpha\beta\lambda^2 & \alpha\beta\lambda^2 - 1 & -\beta\lambda \\ -\alpha\lambda & -\alpha\lambda & -1 \end{pmatrix} \begin{pmatrix} 1 & 0 \\ 0 & 1 \\ 0 & 0 \end{pmatrix} \\ &= \frac{1}{2\alpha\beta\lambda^2 - 1} \begin{pmatrix} \alpha\beta\lambda^2 - 1 & -\alpha\beta\lambda^2 \\ -\alpha\beta\lambda^2 & \alpha\beta\lambda^2 - 1 \\ -\alpha\lambda & -\alpha\lambda \end{pmatrix}. \end{aligned}$$

Choosing $\alpha = \beta = \lambda = \frac{1}{2}$, we obtain

$$W = \frac{1}{14} \begin{pmatrix} 15 & 1 \\ 1 & 15 \\ 4 & 4 \end{pmatrix},$$

indicating that both compounds contribute feature information to the reaction (i.e. being related to the compounds glucose 6-phosphate and fructose 6-phosphate). The inferred feature vectors for both species do now contain information about the other species' features, indicating that a species carrying a corresponding annotation will be close to them in the reaction network.

When comparing two models of the phosphoglucoisomerase reaction by the similarities inferred from feature propagation (as described by Eq. (6) and setting $S = I$), we end up with

$$\begin{aligned} \psi^{\mathrm{fp}} &= \begin{pmatrix} \psi^{\mathrm{fp}}_{ap} & \psi^{\mathrm{fp}}_{aq} & \psi^{\mathrm{fp}}_{ay} \\ \psi^{\mathrm{fp}}_{bp} & \psi^{\mathrm{fp}}_{bq} & \psi^{\mathrm{fp}}_{by} \\ \psi^{\mathrm{fp}}_{xp} & \psi^{\mathrm{fp}}_{xq} & \psi^{\mathrm{fp}}_{xy} \end{pmatrix} \\ &= \begin{pmatrix} 1 & \frac{15}{113} & \frac{8}{\sqrt{113}} \\ \frac{15}{113} & 1 & \frac{8}{\sqrt{113}} \\ \frac{8}{\sqrt{113}} & \frac{8}{\sqrt{113}} & 1 \end{pmatrix}. \end{aligned}$$

In comparison to the original similarities (see Eq. (1); assuming $\sigma_{ab} = 0$)

$$\sigma = \begin{pmatrix} 1 & 0 & 0 \\ 0 & 1 & 0 \\ 0 & 0 & 0 \end{pmatrix},$$

this matrix indicates that glucose 6-phosphate, fructose 6-phosphate, and the reaction between them should be matched even though the reaction has not been annotated in both models. The off-diagonal elements show that we have to face a certain "background similarity" because all the elements in the model become associated with all features. The different weighting of the features in the propagated vectors is responsible for the difference between the similarity values.

5.2 Similarity propagation

Next, we calculate the propagated similarities

$$\psi^{\mathrm{sp}} \quad = \quad (I - \lambda Q)^{-1} \, \sigma.$$

We consider only the pairs in the following vector and assume that $\sigma_{aq} = \sigma_{bp} = 0$ because the species elements contain totally different annotations and that $\sigma_{xy} = 0$ because the reactions contain no annotations at all. If we insert this and only consider the relevant part of the 9×9 matrix Q, the propagated similarities read

$$\begin{pmatrix} \psi_{ap} \\ \psi_{aq} \\ \psi_{bp} \\ \psi_{bq} \\ \psi_{xy} \end{pmatrix} = \left(I - \lambda \begin{pmatrix} 0 & 0 & 0 & 0 & \beta^2 \\ 0 & 0 & 0 & 0 & \beta^2 \\ 0 & 0 & 0 & 0 & \beta^2 \\ 0 & 0 & 0 & 0 & \beta^2 \\ \alpha^2 & \alpha^2 & \alpha^2 & \alpha^2 & 0 \end{pmatrix} \right)^{-1} \begin{pmatrix} \sigma_{ap} \\ 0 \\ 0 \\ \sigma_{bq} \\ 0 \end{pmatrix}$$

$$= \begin{pmatrix} 1 & 0 & 0 & 0 & -\lambda\beta^2 \\ 0 & 1 & 0 & 0 & -\lambda\beta^2 \\ 0 & 0 & 1 & 0 & -\lambda\beta^2 \\ 0 & 0 & 0 & 1 & -\lambda\beta^2 \\ -\lambda\alpha^2 & -\lambda\alpha^2 & -\lambda\alpha^2 & -\lambda\alpha^2 & 1 \end{pmatrix}^{-1} \begin{pmatrix} \sigma_{ap} \\ 0 \\ 0 \\ \sigma_{bq} \\ 0 \end{pmatrix}$$

$$= \frac{1}{4\alpha^2\beta^2\lambda^2 - 1}$$

$$\cdot \begin{pmatrix} 3\alpha^2\beta^2\lambda^2 - 1 & -\alpha^2\beta^2\lambda^2 & -\alpha^2\beta^2\lambda^2 & -\alpha^2\beta^2\lambda^2 & -\beta^2\lambda \\ -\alpha^2\beta^2\lambda^2 & 3\alpha^2\beta^2\lambda^2 - 1 & -\alpha^2\beta^2\lambda^2 & -\alpha^2\beta^2\lambda^2 & -\beta^2\lambda \\ -\alpha^2\beta^2\lambda^2 & -\alpha^2\beta^2\lambda^2 & 3\alpha^2\beta^2\lambda^2 - 1 & -\alpha^2\beta^2\lambda^2 & -\beta^2\lambda \\ -\alpha^2\beta^2\lambda^2 & -\alpha^2\beta^2\lambda^2 & -\alpha^2\beta^2\lambda^2 & 3\alpha^2\beta^2\lambda^2 - 1 & -\beta^2\lambda \\ -\alpha^2\lambda & -\alpha^2\lambda & -\alpha^2\lambda & -\alpha^2\lambda & -1 \end{pmatrix}$$

$$\cdot \begin{pmatrix} \sigma_{ap} \\ 0 \\ 0 \\ \sigma_{bq} \\ 0 \end{pmatrix}$$

$$= \frac{1}{4\alpha^2\beta^2\lambda^2 - 1} \begin{pmatrix} 3\alpha^2\beta^2\lambda^2 - 1 & -\alpha^2\beta^2\lambda^2 \\ -\alpha^2\beta^2\lambda^2 & -\alpha^2\beta^2\lambda^2 \\ -\alpha^2\beta^2\lambda^2 & -\alpha^2\beta^2\lambda^2 \\ -\alpha^2\beta^2\lambda^2 & 3\alpha^2\beta^2\lambda^2 - 1 \\ -\alpha^2\lambda & -\alpha^2\lambda \end{pmatrix} \begin{pmatrix} \sigma_{ap} \\ \sigma_{bq} \end{pmatrix}.$$

With $\alpha = \beta = \lambda = \frac{1}{2}$ we obtain

$$\begin{pmatrix} \psi_{ap} \\ \psi_{aq} \\ \psi_{bp} \\ \psi_{bq} \\ \psi_{xy} \end{pmatrix} = \frac{1}{60} \begin{pmatrix} 61 & 1 \\ 1 & 1 \\ 1 & 1 \\ 1 & 61 \\ 8 & 8 \end{pmatrix} \begin{pmatrix} \sigma_{ap} \\ \sigma_{bq} \end{pmatrix}.$$

In this example, a matching based on similarity propagation will always favour the pairs (ap), (bq), and (xy), as long as the similarities σ_{ap} and σ_{bq} are positive. As in the case of feature

propagation, also unrelated pairs are assigned a small "background similarity", which depends on the propagation parameters. This background similarity is now smaller, but also the similarity of the correct pair (xy) ($\psi_{xy} = \frac{4}{15}$ for $\sigma_{ap} = \sigma_{bq} = 1$) does not stand out as clearly as before. Furthermore, the similarities ψ_{ap} and ψ_{bq} can assume values larger than 1. To obtain values in the range $[0, 1]$, we need to normalise the similarities as explained in Section 3.

6 Semantic propagation through cofactors

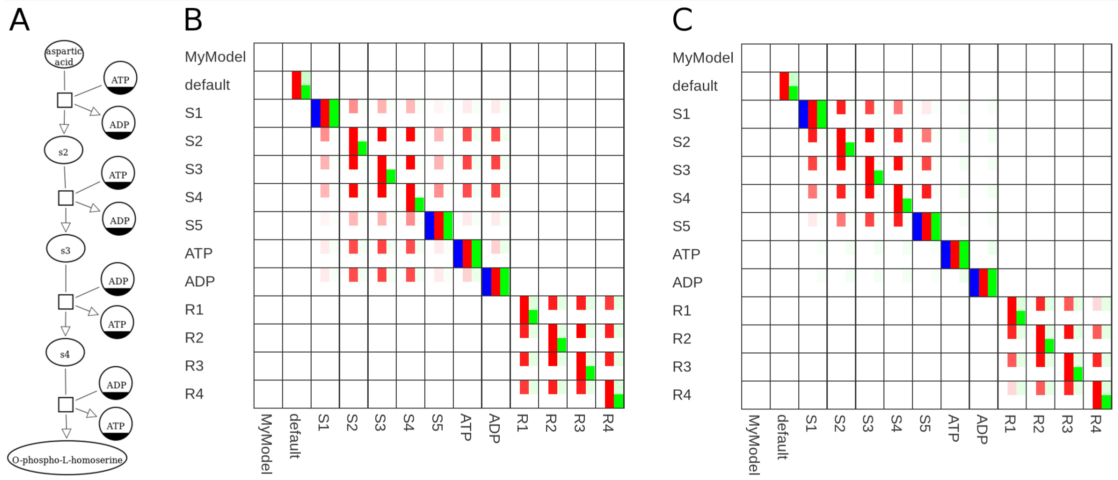

Figure S2: Comparison of two different alignments, with and without propagation through cofactors. We consider a linear reaction chain in which each reaction involves the same cofactors (A). This model is aligned to itself like in the analysis in Figure 3. In this example, however, not only the first and the last species in the chain are annotated but also the two cofactors. Similarities between the elements of the model have been computed with (B) and without (C) propagation through cofactors. The matrices show the pairwise similarities (top) and the matching based on these similarities (bottom) for the direct similarity measure (blue), the propagated feature (red), and the propagated similarities (green). The propagation along cofactors leads to an increase in the "background" similarity, the non-specific similarity of unrelated elements as seen between S2-S4 and R1-R4.

In metabolic and signalling pathways, cofactors act as hubs connecting distant reactions. In network visualisation's cofactors are thus often split into separate nodes connected to single reactions in order to get a clearer picture of the pathway structure (e.g. see Figure S2). Given that cofactors do not necessarily contribute to a pathway's structure (as we interpret it), we should investigate whether it makes sense to propagate semantic information through them.

In general, propagation along cofactors can lead to the problem that unrelated reactions inherit semantic information from each other. This in turn leads to higher background similarities which could result in erroneous model alignments. An example of this phenomenon can be found in Figure S2: annotated cofactors increase the general similarity of all elements in the linear chain, making it harder to match them unambiguously.

For this reason, we have compiled a list of annotations for cofactors in Table 1, defining a set of metabolites along which no propagation is carried out in our tools ($\rho_{ax} = \rho_{xa} = 0$ for all cofactors a). This idea increases the specificity of our method across all possible applications.

Name	KEGG Compound ID	ChEBI ID
H_2O	C00001	CHEBI:15377
ATP	C00002	CHEBI:15422
NAD	C00003	CHEBI:15846
NADH	C00004	CHEBI:16908
NADPH	C00005	CHEBI:16474
NADP	C00006	CHEBI:18009
O_2	C00007	
ADP	C00008	CHEBI:16761
Orthophosphate	C00009	CHEBI:18367
CO_2	C00011	CHEBI:16526
Diphosphate	C00013	CHEBI:18361
Protein	C00017	CHEBI:36080
AMP	C00020	CHEBI:16027
Iron	C00023	CHEBI:24875
GDP	C00035	CHEBI:17552
GTP	C00044	CHEBI:15996
RNA	C00046	
Ca^{2+}	C00076	CHEBI:29108
H^+	C00080	CHEBI:24636
GMP	C00144	CHEBI:17345
Phosphoprotein	C00562	
cAMP	C00575	CHEBI:17489
mRNA		CHEBI:33699

Table 1: cofactors along which no information is propagated.

7 Comparing alignment quality after removal of annotations from different types of nodes

In a further analysis we investigated whether it is harder to match models lacking annotations only on species or only on reactions. To answer this question we have repeated in the analysis performed in Figure 5. In this analysis random annotations have been removed from one model, which was then aligned to a second model. In Figure 3 we show the effects of random removal of annotations from either species or reactions in the first model, i.e. the precision and the recall of the matching.

The evaluation after the removal of reaction annotations, on the one hand, lead to similar results as in the main text. The quality of the matching (precision as well as recall) became worse for larger numbers of removed annotations in a linear manner. As shown in the main text, the propagated similarities produce the best matching while the propagated features and the direct similarities performed worse.

On the other hand, the removal of species annotations led to an unexpected new behaviour. First of all, the quality of the alignment is better when only species annotations are removed. Probably, the reason is that the information in the reaction annotations is more relevant to the correct alignment of these two MAP kinase cascades because more distinct annotations are used. Second, ambiguities in the matching of certain species exist, as these species have the same annotations. If only some of these annotations are removed, the greedy matching might prefer to match elements incorrectly as they contain the remaining annotations. When most of the species annotations are removed, the matching is not influenced by the ambiguous annotations anymore

and its quality is improving again.

To investigate whether this behaviour really stems from ambiguities in species annotations, which is recovered by available reaction annotations, we have performed the analyses from Figure S3 again. The only difference is that before species or reaction annotations are partially removed, the annotations on the other type of nodes is erased from the model. As seen in Figure S4, the non-linear trend from the removal of species annotations with reaction annotations being present has disappeared, which underlines our hypothesis. The results in fact show the same linear trend as expected from the analysis in Figure 5 and from the removal of reaction annotations with species annotations being present.

8 Alignment of models described to different detail

As mentioned in Section 2, the two propagation schemes may produce different results when they are used to compare models containing lumped reactions. We have evaluated this behaviour in detail for a large set of different models which are variants of a linear chain containing lumped species and reactions (see Figure S5).

For all pairs of these models we have computed alignment matrices (Figure S6). A general observation from this analysis is that the lumping only affects the quality of the alignment in the direct vicinity of the lumped model elements. Nevertheless, there are some differences in the alignments based on the different similarities. The direct similarity measure has no particular preference in the mapping of a lumped element to its detailed counterparts (e.g. in the alignment of the models MB and MD). Except for cases in which an unambiguous mapping is enforced by the annotations (i.e. when one detailed element is annotated using more distinct annotations than the other), the alignment occurs randomly.

The alignments based on feature propagation and similarity propagation behave differently, as they both have a certain preference for elements they regard as most similar. An alignment based on feature propagation tends to map a lumped element to the center of the corresponding detailed elements (e.g. mapping S5 to S5 when aligning the models MB and MD). This behaviour can be explained by the fact that the similarity based on propagated features tries to map elements such that they have a similar "mix" of inherited features. In contrast, similarity propagation will mostly map the lumped element to one of the outmost detailed elements. This behaviour is driven by the fact that similarity propagation tends to reduce the number of gaps in a model alignment.

Apart from these general observations, we have also experienced some interesting behaviour in the alignment based on similarity propagation. The alignments of the models MB and MH, MD and MF, and MF and MH show an erroneous matching of the annotated lumped components and the elements around them, which cannot easily be explained. Nevertheless, the model alignment based on propagated semantic information performs quite well and, in most cases, feature propagation performs a little better than similarity propagation.

9 Using KEGG as a reference database for annotation prediction

The prediction of new annotations for model elements is based on an alignment of the model to a big, annotated pathway map. After having aligned the model to the pathway map, annotations can be transferred from elements in the map to the corresponding elements in the model. In the main text the pathway map has been taken from BioModels Database, but in principle any collection of annotated models could serve as such. To compare the quality of the annotation predictions based

on different pathway maps, we have rerun the analysis shown in Figure 6 using KEGG Pathways instead of BioModels Database.

As shown in Figure S7, the results differ only slightly between the two model resources when they are applied to predict annotations in a metabolic network. Thus, the fact that BioModels Database contains manually curated annotations does not improve the quality of the model annotations. Since the annotation quality does not matter significantly, we plan to increase the coverage of the background model by incorporating as many annotated models to it as possible in the future.

Removing species annotations:

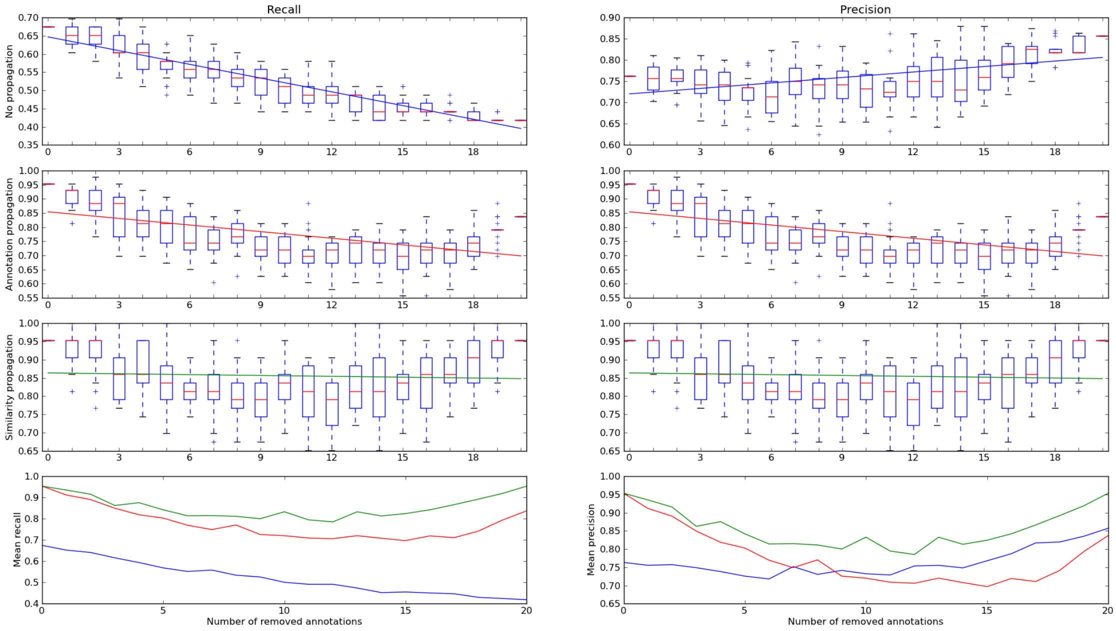

Removing reaction annotations:

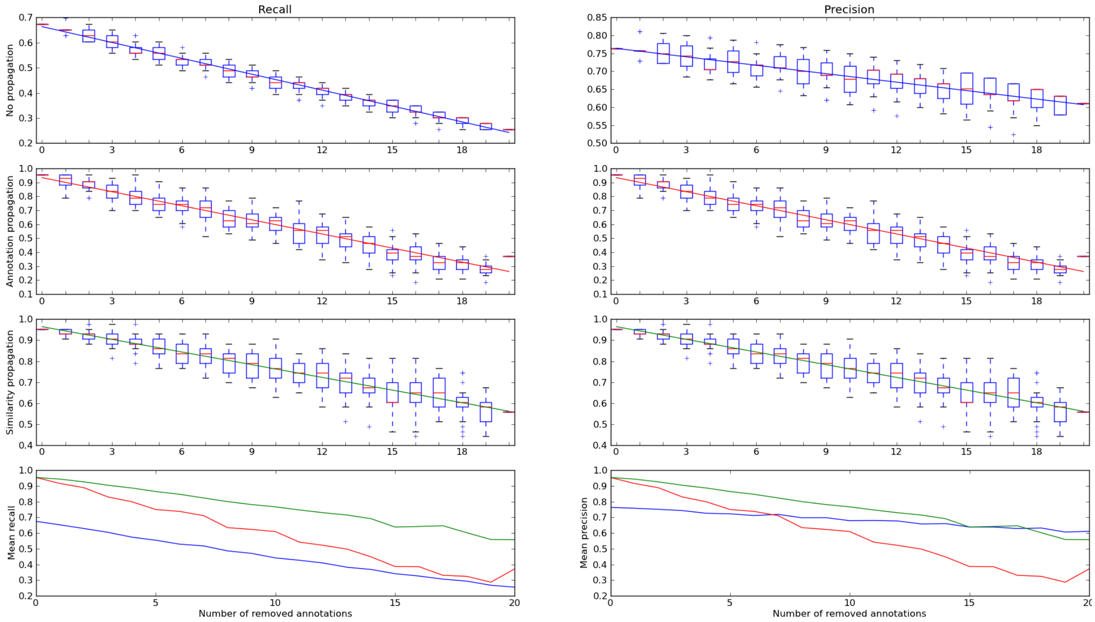

Figure S3: Assessing the quality of model alignments. This analysis corresponds to the analysis performed in Figure 5. The BioModels 9 and 11 were aligned after a number of randomly chosen annotations had been removed repeatedly from BioModel 9. Recall and precision of the matching based on the direct similarity, the propagated features, and the propagated similarities are compared. The analysis from the main text has been conducted again, focusing on the removal of either only species annotations (top) or reaction annotations (bottom). While the removal of reaction annotations leads to a steady decline in the quality of the matching, the removal of species has a non-linear effect on recall and precision.

Removing species annotations (no reaction annotations present):

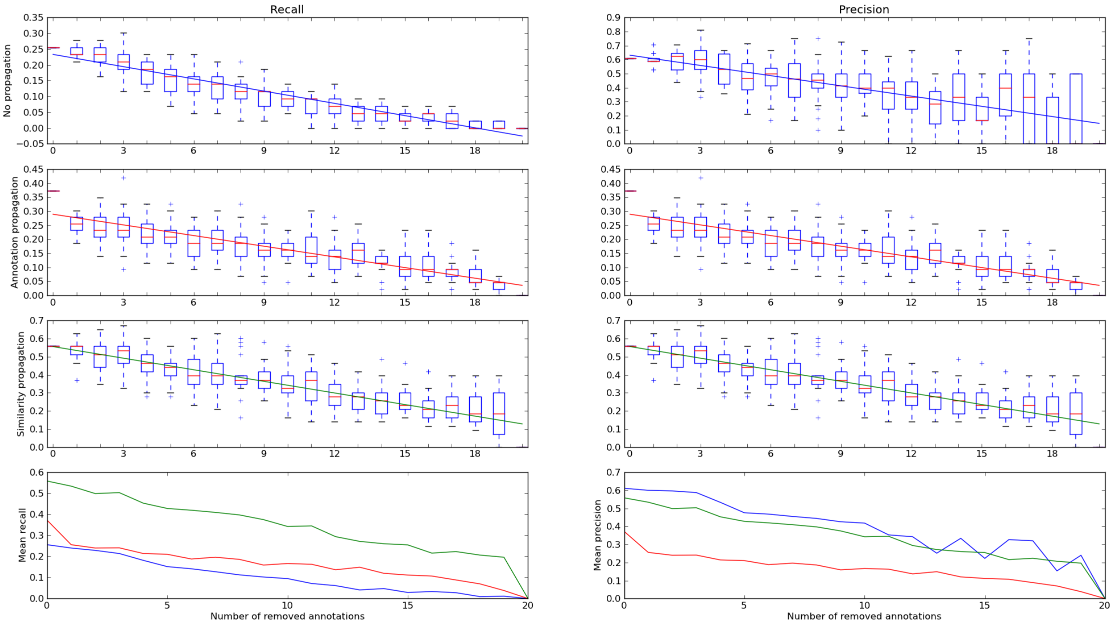

Removing reaction annotations (no species annotations present):

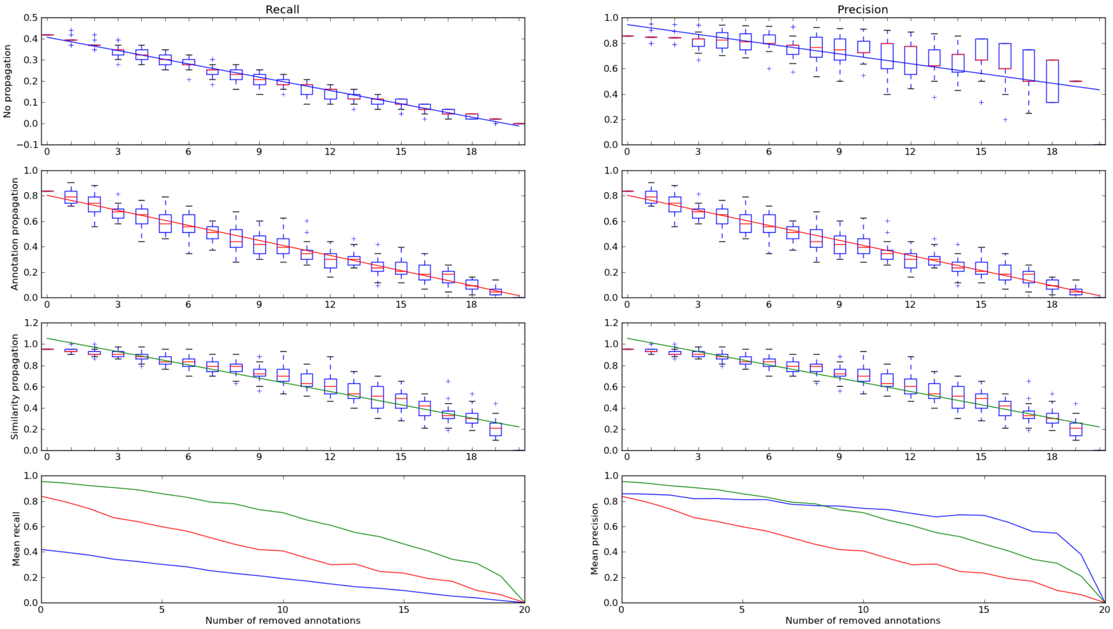

Figure S4: Similar analysis as in Figure S3. Before species or reaction annotations were partially removed in the analysis, all reaction or species annotations, respectively, had been removed.

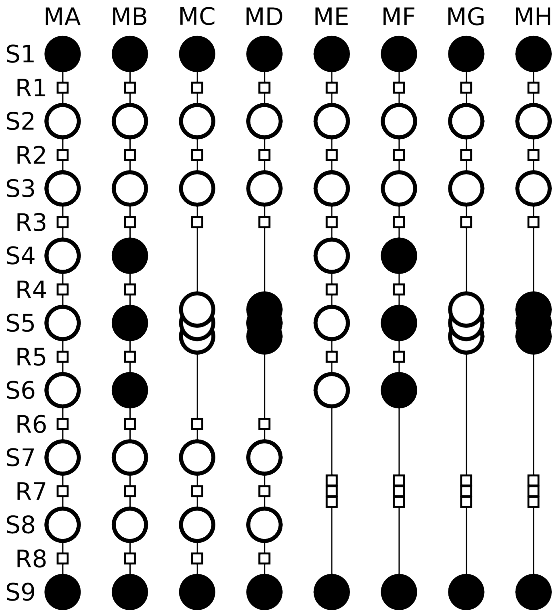

Figure S5: Models used to analyse the effects of lumped species and reactions on the performance of the matching based on the different similarities. All models represent linear reaction chains of species (circles) and reactions (squares) which are lumped (superimposed nodes) or annotated (filled circles) at different positions.

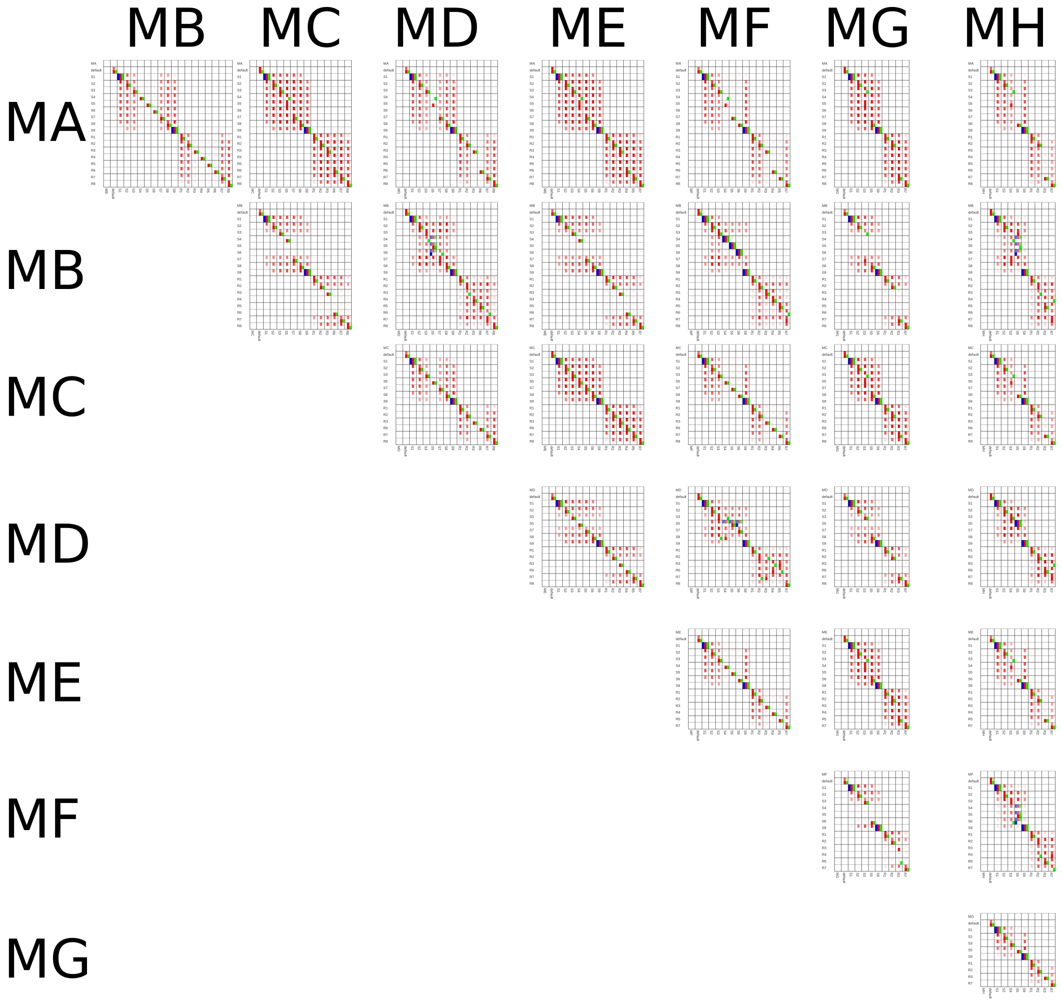

Figure S6: Individual alignments of the models presented in Figure S5. The matrix consists of alignments of model pairs as they are depicted in Figure S2. Each of the alignments shows the similarities of the model's element pairs for the direct similarity measure, the propagated features, and the propagated similarities and the alignments based on these measure.

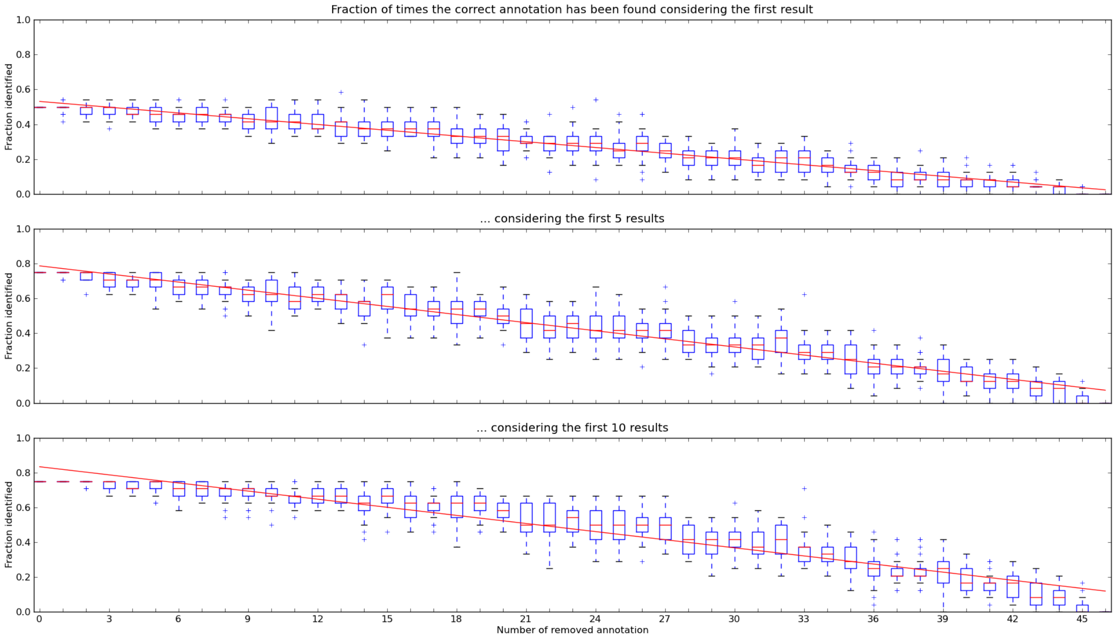

Figure S7: Quality of annotation predictions in BioModel 61 after random removal of annotations. The same analysis is shown in Figure 6. The only difference is that this prediction has been made using the KEGG Pathway maps instead of BioModels as a background data set for annotation prediction.
